# Partial weight reduction protocols in cats lead to better weight outcomes, compared with complete protocols, in cats with obesity

**DOI:** 10.3389/fvets.2023.1211543

**Published:** 2023-06-20

**Authors:** Alexander J. German, Georgiana R. T. Woods-Lee, Vincent Biourge, John Flanagan

**Affiliations:** ^1^Institute of Life Course and Medical Sciences, Faculty of Health and Life Sciences, University of Liverpool, Liverpool, United Kingdom; ^2^Royal Canin Research Centre, Aimargues, France

**Keywords:** weight management, feline, nutrition, overweight, weight loss, body composition, essential nutrients

## Abstract

**Background:**

To date, there have been no studies comparing outcomes of cats with obesity following either complete or partial weight reduction protocols.

**Methods:**

Fifty-eight cats participated in this non-randomized observational cohort study, including 46 (79%) and 12 (21%) that underwent complete or partial weight reduction protocols, respectively. Weight loss outcomes, body composition changes and essential nutrient intake were compared between cats in the two groups.

**Results:**

All cats remained healthy, and those on a complete weight reduction protocol lost a median of 23% (range 10–39%) of starting body weight (SBW) over 294 days (113–967 days), whereas those undergoing partial restriction lost 25% (10–41%) over 178 days (54–512 days). Neither duration nor percentage weight loss differed between groups, but those that followed a partial weight reduction protocol lost weight at a faster rate (0.81% per week) and required fewer visits (4–19) than those that followed a complete weight reduction protocol (0.61% per week, *p* = 0.028; 11, 4–40 visits, *p* = 0.009). Further, lean tissue mass declined in cats on a complete weight reduction protocol (pre: 4.20 kg, 2.64–5.72 kg; post: 3.90 kg, 2.76–5.24 kg, *p* < 0.001), whereas lean tissue mass was unchanged in cats on partial weight reduction protocols (pre: 3.45 kg, 2.79–4.71 kg; post: 3.41 kg, 2.90–4.59 kg, *p* = 0.109). In 33 (57%) cats, median intake of selenium per day was less than NRC AI and RA recommendations, whilst intake was under FEDIAF recommendation in 42 (72%) cats. Median intake of choline per day was less than NRC MR and RA recommendations in 22 (38%) and 53 (91%) cats, respectively, whereas it was under the FEDIAF recommendation in 51 (88%) cats. In a small proportion (12–14%) of cats, phenylalanine/tyrosine and potassium were under recommendations; besides these, no other essential nutrient deficiencies were seen, and there were no differences between cats undergoing complete and partial weight reduction.

**Conclusion:**

Partial weight reduction protocols in cats lead to quicker average weight loss, with the possibility that lean tissue loss might be minimized. Such protocols might be more suitable for older cats and those with marked obesity.

## 1. Introduction

Feline obesity is a prevalent chronic disease with adverse effects on both health and welfare (1). Controlled weight reduction is most-commonly used, comprising dietary caloric restriction and altered physical activity principally through play (2, 3). It is recommended that such protocols be tailored to the individual, with target weight being adapted according to clinical and other factors (4). ‘Complete’ weight reduction protocols are designed with the target of the animal reaching ideal weight (4), but such an approach increases the potential for failure, because duration of the protocol is associated both with a slowing in rate of weight loss and an increase in non-compliance (4). Conversely, ‘partial’ weight reduction protocols involve deliberately planning for the animal to lose only a portion of the excess weight, meaning that the target weight for the end point of the weight reduction phase is above the animal’s ideal weight. This approach has the key advantage of the target weight being more achievable, taking weeks rather than months; despite remaining above ideal weight, both functional improvements and improved quality of life still occur (5, 6). However, partial weight reduction protocols suffer the main disadvantage of the animal remaining in overweight condition, such that any weight loss benefits are likely to be less marked than with complete weight reduction. To the authors’ knowledge, no previous studies have compared differences between complete and partial weight reduction protocols.

Whether a complete or a partial weight reduction protocol is chosen, dietary caloric restriction usually requires the feeding of a therapeutic diet. Such diets are formulated with increased amounts of essential nutrients relative to their energy content, to ensure that essential requirements are met even when feeding less than metabolisable energy requirements required for maintenance (MER). Internationally-accepted recommendations on daily essential nutrient intake are available for cats, including those of the National Research Council (7) and the European Pet Food Industry Federation (Fédération Européenne de l’Industrie des Aliments pour Animaux Familiers; FEDIAF) (8). However, there has previously been little research into adequacy of essential nutrients in animals with obesity that undergo controlled weight reduction using a therapeutic diet. To the authors’ knowledge, there have been only two studies assessing essential nutrient intake based upon reported food intake during a weight reduction period (9, 10). In the first study, 17 overweight cats underwent weight reduction for 8 weeks utilizing reduced-energy maintenance diets, and with animals consuming approximately 80% of maintenance energy requirements (9). Median daily intake of all essential nutrients was above either NRC 2006 minimal requirement (MR) or NRC 2006 adequate intake (AI, when no MR had been demonstrated), except for the intake of selenium which, in 4 cats, was marginally under NRC 2006 AI. All cats in this study remained in good health and did not show any signs of essential nutrient deficiencies. In the second study, cats with obesity were fed a therapeutic weight reduction diet for 10 weeks, during which time they lost a mean of 0.9% of their body weight per week (10). Again, all cats remained healthy, but the average intakes of both arginine and choline were less than the NRC 2006 AI in most cats, whilst borderline intake of phenylalanine and tyrosine was reported in some cats. A key limitation of both studies was the fact that they were short term with cats only losing a modest amount of weight (<10%). They also did not compare intakes of cats that underwent ‘complete’ versus ‘partial’ weight reduction; since such protocols are likely to differ in length of time that the cat must undergo caloric restriction, the risk of nutrient deficiency might plausibly differ between them.

Therefore, the aim of the current was to compare outcomes in cats undergoing complete versus partial weight reduction protocols, in terms of various outcomes including rate of weight loss, changes in body composition. Additional aims included determining the adequacy of essential nutrient intake cats undergoing either complete or partial weight reduction protocols, and to determine whether any differences existed between them.

## 2. Materials and methods

### 2.1. Study cats, eligibility criteria, and ethical considerations

This study involved adult pet cats with obesity that attended the Royal Canin Weight Management Clinic, University of Liverpool, UK, between January 2005 and July 2021. Eligible cats had completed their period of weight reduction protocol by February 2022, meaning that they had reached the target weight that had been set for them in advance of their program. Cats were not eligible if they had comorbidities [e.g., endocrine disease (including diabetes mellitus and acromegaly), hepatic disease, renal disease, and gastrointestinal disease] that might affect their food intake or nutritional requirements. Cats had to have been fed a therapeutic weight reduction diet (see below), which was either just dry therapeutic food or a mixture of dry and wet food. Switching between formulations was not permitted. Finally, use of other foods or significant dietary non-compliance was also not allowed (as determined by the owner diary used for weight reduction; see below).

The University of Liverpool Veterinary Research Ethics Committee (RETH000353 and VREC793), the WALTHAM ethical review committee and the Royal Canin Ethical Review Committee (150720–55) all approved the study. As part of the University approval, the nature of the procedures performed were considered and, specifically, whether they should be classified as experimental procedures. In this respect, all clinical procedures were conducted in accordance with relevant guidelines (e.g., standard operating procedures) and regulations. Further, the foods used in the study were commercially-available therapeutic diets (already commonly used by veterinarians to manage obesity) and were fed for the clinical benefit of the cats in the study. As a result, in the ethical approvals granted (RETH000353 and VREC793), neither the clinical procedures used nor the clinical use of the therapeutic diets were deemed to involve animal experimentation and, therefore, fell outside the remit of national legislation (e.g., the revised Animals [Scientific Procedures] Act 1986).

The owners of all cats that participated gave their informed consent in writing, for clinical management of their cat’s obesity, for the use of anonymized data and surplus clinical samples for research purposes, and for use of data for publication. All procedures performed were clinical in nature and undertaken for the benefit of the animal.

### 2.2. Definitions and study groups

For the purposes of this study, the ‘ideal weight’ of the patient was defined as the weight at which the cat’s body fat mass was optimal and was either determined by body composition data from dual-energy X-ray absorptiometry (DEXA) or by body condition score (BCS, see below). In contrast, ‘target weight’ was defined as the goal weight set for the period of controlled weight reduction, and this was individualized for each cat (see below).

Cats were grouped according to whether they underwent a complete or partial weight reduction protocol. In a complete weight reduction protocol, the primary aim was to return the cat to within ±5% of their ideal weight; to achieve this, the chosen target weight for the cat was the same as the ideal weight. In contrast, for a partial weight reduction protocol, the target weight was deliberately set to be greater than the ideal weight (4). This meant that cats undergoing partial weight reduction would still be in overweight body condition (i.e., at least 10% above their ideal weight) at the end of their protocol; however, the intention was for them to have lost enough weight to improve their health and wellbeing; for example, weight loss of 10 ± 6.3% of starting weight was sufficient to improve mobility and quality of life, as observed by owners (6). Reasons for choosing a partial weight reduction protocol were age (i.e., >9 years, 3 cats), marked obesity (i.e., >40% above ideal weight; 2 cats) and both age and marked obesity (7 cats). Marked obesity was considered because it is known to be negatively associated with outcome of weight reduction in cats (11), whilst age was considered because of the increased risk of having other chronic diseases (12).

### 2.3. Initial assessment

Before the weight reduction protocol was implemented, the health of all cats was assessed using physical examination, routine hematology, serum biochemistry and urinalysis. In all cats, BCS was measured (11) and, in most, body composition was measured by DEXA as previously described (2). At this stage, either body composition data or BCS were used to set the ideal weight, as previously described (6, 11). Briefly, for body composition data, measurements of lean mass, fat mass and bone mineral content (in grams) were keyed into an electronic spreadsheet (Excel for Mac, version 16.71, Microsoft), which used a bespoke mathematical formula to predict body composition after weight reduction, with optimal body fat mass assumed to be ~20% for cats (2, 13, 14). This mathematical formula incorporated information on known body composition alterations from previous studies using the same DEXA machine and software (2, 15). For cats where body composition data were not available, ideal weight was instead determined from BCS (6); briefly, the starting weight of the cat was divided by 1.2, 1.3 or 1.4, for cats whose BCS of 7, 8, and 9, respectively. Clinical information was used to set the target weight and decide whether the cat would undergo complete or partial weight reduction (see above).

### 2.4. Weight reduction period

Details of our feline weight reduction regimen have previously been published (2, 11). Each protocol was individualized to the cat and involved feeding one or more therapeutic diet (Table 1) for encouraging controlled weight loss, all made by the same company (Royal Canin SAS, Aimargues, France). There were two dry diets (D1: Obesity Management; D2 Satiety Support) and two wet diets (W1: Obesity Management; W2: Satiety Support). Daily food portions were weighed out by owners using electronic kitchen scales; to be certain that all scales were accurate, the first portion was weighed on the clinic’s own calibrated electronic scales (Salter, Tonbridge, UK), and the owner took this portion home to weigh on their own scales. In addition, owners were given advice on how to stimulate physical activity, for example, using toys to encourage play. During the program, owners maintained a diary where they recorded diet ration fed, activity undertaken, periods of illness and any dietary non-compliance. Each cat was rechecked approximately every 2–4 weeks; on each occasion, bodyweight was measured, the diary records scrutinized, and adjustments being made to their feeding protocol if deemed to be necessary (2).

**Table 1:** Average composition of the therapeutic diets used for weight reduction in 53 cats with obesity.

Criterion	Dry diet 1^1^		Dry diet 2^2^		Wet diet 1^3^		Wet diet 2^4^	
ME content	3,394 kcal/kg		2,963 kcal/kg		620 kcal/kg		677 kcal/kg	
	As fed ^5^	Per 1,000 kcal	As fed ^5^	Per 1,000 kcal	As fed ^5^	Per 1,000 kcal	As fed ^5^	Per 1,000 kcal
Moisture	9.9	29	9.8	33	83.7	1,352	83.9	1,237
Crude protein	40.2	118	32.6	110	7.6	120	7.4	112
Crude fat	9.7	29	8.7	29	1.9	31	2.3	33
Crude fiber	6.3	19	13.5	46	1.4	23	1.2	17
Total dietary fiber	13.0	38	21.6	73	1.6	26	1.4	21
Ash	7.6	22	8.1	27	1.6	25	1.7	25
Arginine (g)	2.16	6.4	1.75	5.9	0.42	6.8	0.45	6.7
Histidine (g)	0.79	2.3	0.63	2.1	0.17	2.7	0.18	2.7
Isoleucine (g)	1.48	4.3	1.17	4.0	0.26	4.3	0.28	4.1
Methionine (g)	0.73	2.2	0.59	2.0	0.17	2.8	0.17	2.5
Met and cys (g)	1.32	3.9	1.03	3.5	0.30	4.9	0.30	4.5
Leucine (g)	3.08	9.1	2.48	8.4	0.55	8.9	0.59	8.8
Lysine (g)	1.71	5.0	1.22	4.1	0.48	7.7	0.50	7.4
Phenylalanine (g)	1.59	4.7	1.37	4.6	0.31	5.1	0.34	5.0
Phe and Tyr (g)	2.85	8.4	2.37	8.0	0.57	9.2	0.59	8.8
Threonine (g)	1.39	4.1	1.13	3.8	0.30	4.8	0.32	4.8
Tryptophan (g)	0.37	1.1	0.30	1.0	0.08	1.4	0.09	1.4
Valine (g)	1.77	5.2	1.42	4.8	0.37	5.9	0.40	5.8
Taurine (g)	0.19	0.6	0.24	0.8	0.17	2.8	0.13	1.9
Linoleic acid (g)	2.01	5.94	1.71	5.79	0.32	5.14	0.43	6.33
Arachidonic acid (g)	0.08	0.23	0.07	0.24	0.04	0.57	0.04	0.66
EPA and DHA (g)	0.15	0.45	0.16	0.54	0.01	0.10	0.01	0.12
Calcium (g)	1.26	3.70	1.26	4.27	0.25	3.98	0.27	3.96
Phosphorus (g)	1.15	3.39	1.09	3.66	0.21	3.36	0.21	3.16
Magnesium (mg)	86	253	76	257	14	220	12	175
Sodium (mg)	484	1,425	542	1828	166	2,673	160	2,370
Potassium (g)	0.98	2.90	1.03	3.47	0.18	2.82	0.17	2.46
Chloride (mg)	953	2,808	1,058	3,571	189	3,046	163	2,412
Iron (mg)	16.8	49.4	14.3	48.4	3.1	49.6	3.1	46.4
Copper (mg)	1.6	4.7	1.7	5.7	0.4	6.5	0.3	4.5
Zinc (mg)	17.6	51.8	17.4	58.7	2.9	47.4	3.2	47.6
Manganese (mg)	6.2	18.2	5.7	19.4	0.3	4.8	0.3	4.9
Selenium (μg)	36	105	34	116	9	137	10	155
Iodine (μg)	354	1,044	360	1,215	42	675	33	491
Vitamin A (RE)	786	2,316	819	2,765	1,585	25,548	1,377	20,343
Vitamin D3 (μg)	2.3	6.9	2.4	8.0	0.6	9.1	0.7	9.7
Vitamin E (mg)	700	2062	691	2,331	71	1,139	80	1,183
Thiamine (mg)	1.62	4.77	1.65	5.57	2.39	38.51	1.34	19.73
Riboflavin (mg)	6.4	18.8	6.1	20.7	0.2	2.5	0.2	2.5
Pyridoxine (mg)	4.5	13.2	4.5	15.3	0.2	2.7	0.2	3.0
Niacin (mg)	19.1	56.4	20.0	67.7	2.0	32.6	2.6	38.5
Pantothenic acid (mg)	6.0	17.8	6.3	21.2	0.5	8.4	0.6	8.8
Cobalamin (μg)	16.6	49.0	16.9	57.2	1.1	17.0	1.4	21.2
Folic acid (μg)	1,109	3,267	2,120	7,155	51	824	57	846
Biotin (μg)	336	989	339	1,143	7.5	121	6.5	97
Choline (mg)	302	891	314	1,060	49	787	64	947

^1^Obesity Management, Royal Canin; ^2^Satiety Weight Management, Royal Canin; ^3^Obesity Management, Royal Canin; ^4^Satiety Weight Management, Royal Canin; ^5^expressed as grams per 100 g; DHA, docosahexanoic acid; DM, dry matter; EPA, eicosapentanoic acid; ME, metabolisable energy content, calculated using a predictive equation based on TDF; Met and Cys, methionine and cysteine; Phe and Tyr, phenylalanine and tyrosine; TDF: total dietary fiber. ME: metabolisable energy content.

### 2.5. Final assessment

Upon reaching target, all cats were reassessed using the same measures as before (physical examination, routine hematology, serum biochemistry and urinalysis). Again, BCS was performed and, in most cats, DEXA was repeated, which enables body composition changes to be precisely determined (2).

### 2.6. Estimation of essential nutrient intake

The average intake of essential nutrients intake during controlled weight loss was estimated as previously described in a similar canine study (16). Briefly, calculations were based on the average the daily food allocation and the average nutrient content of the therapeutic diet, as reported by the manufacturer (Table 1). If a cat had consumed a mixture of dry and wet food, the nutrient intake was calculated separately for each food, before adding the two added together to determine the overall intake per day. Results are reported as median (range) per kg IBW^0.67^ as well as the number (and percentage) of cats with daily intake of essential nutrients under the recommendations of either the NRC (7) or FEDIAF (8). For NRC, comparisons for each essential nutrient were made either with MR or AI (depending upon what was available), as well was with recommended allowance (RA) were used. Minimal requirement was defined as ‘the minimal concentration of a maximally-bioavailable nutrient that will support a defined physiological state’, whilst AI was defined as ‘the concentration of nutrient demonstrated to support a defined physiological state when no MR has been demonstrated’ (7). Further, RA was either derived from MR after adding a ‘safety factor’ (accounting for both inter-diet variations in bioavailability and inter-individual variations in energy intakes) or derived from AI when no MR was available, i.e., it was observed that animals fed diets containing such amounts did well (7). The FEDIAF recommendations were converted to the same units as used for NRC 2006 to make comparisons simpler.

### 2.7. Data handling and statistical analyses

Data were keyed into a spreadsheet (Microsoft Excel® for Mac version 16.19) and then assessed to ensure that there were no errors, whilst an “online open-access statistical language and environment” (R, version 4.2.3) (17) was used for all statistical manipulations, with level of statistical significance was set at *p* < 0.05, assuming two-sided analyses. Complete datasets were available for all variables except for body composition data because this was not performed in 5 cats both before and after weight loss, and a further 6 cats after weight loss only (Supplementary data); therefore, statistical analyses on body composition data were conducted using data from the remaining 48 cats. All ME intakes are expressed as both kJ and kcal per kg^0.67^ of ideal bodyweight (IBW), whilst weight loss is reported as percentage starting body weight (SBW). Finally, we determined average daily ME intake for weight reduction by first calculating the total ME intake for the weight loss period, and then dividing this by the number of days the protocol lasted.

For the weight loss variables and nutritional intake data, normality was assessed in all sets of continuous data with Shapiro–Wilk tests and by examining Q-Q plots. Given that many datasets were not normally distributed, a decision was made to report continuous results as median (range) and categorical data as absolute numbers and percentages. Wilcoxon signed-rank tests were used to assess differences in body composition measurements before and after weight reduction, whilst differences between cats undergoing complete and partial weight reduction were assessed with the Mann–Whitney test. In both cases, non-parametric effect size was determined by calculating rank biserial (and its 95% confidence interval, 95%-CI) using the ‘effectsize’ package (version 0.7.0) (18). The range of rank biserial values can vary between −1 (all second-sample values greater than all first-sample values) to +1 (all first-sample values greater than all second-sample values), with values of larger (positive or negative) magnitude indicating larger differences between groups. Interpretation was based on the recommendations of Funder and Ozer (19), whereby rank biserials of <0.05, 0.05–0.10, 0.10–0.20, 0.20–0.30, 0.30–0.40 and > 0.40 indicated “tiny,” “very small,” “small,” “medium,” “large” and “very large” effect sizes, respectively.

## 3. Results

### 3.1. Baseline details for study cats

**Figure 1:**
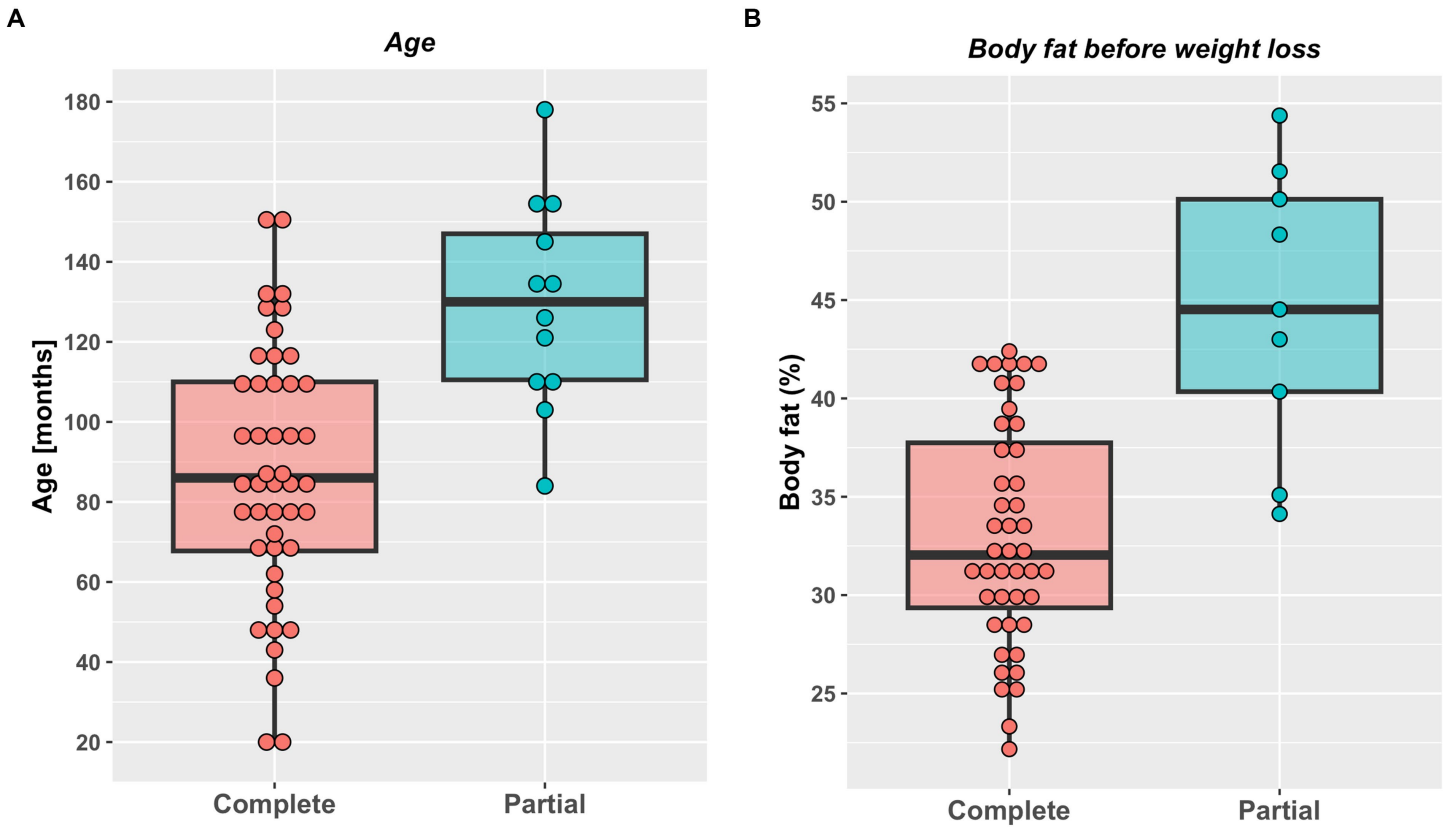
Age **(A)** and initial percentage body fat **(B)** in cats undergoing either complete (46) or partial (12) weight reduction. Red (complete weight reduction protocol) or blue (partial weight reduction protocol) points represent data from individual cats, thick horizontal black lines represent the median of each group, whilst the upper and lower hinges of the boxes represent the inter-quartile range (IQR). The lower whisker represents the smallest observation greater than or equal to the lower hinge of the box minus 1.5 times the IQR; the upper whisker represents the largest observation less than the upper hinge of the box plus 1.5 times the IQR. Cats assigned to complete weight reduction protocols were younger (median 86 months, range 19–151 months) than those assigned to partial weight reduction (median 130 months, range 84–178 months; *p* < 0.001; rank biserial −0.70 [very large effect], 95%-CI -0.84 to −0.46). Percentage body fat was less in cats assigned to complete (median 32%, range 22–42%) compared with partial (median 45%, range 34–54%) weight reduction protocols (*p* < 0.001; rank biserial −0.80 [very large effect], 95%-CI -0.91 to −0.59).

**Figure 2:**
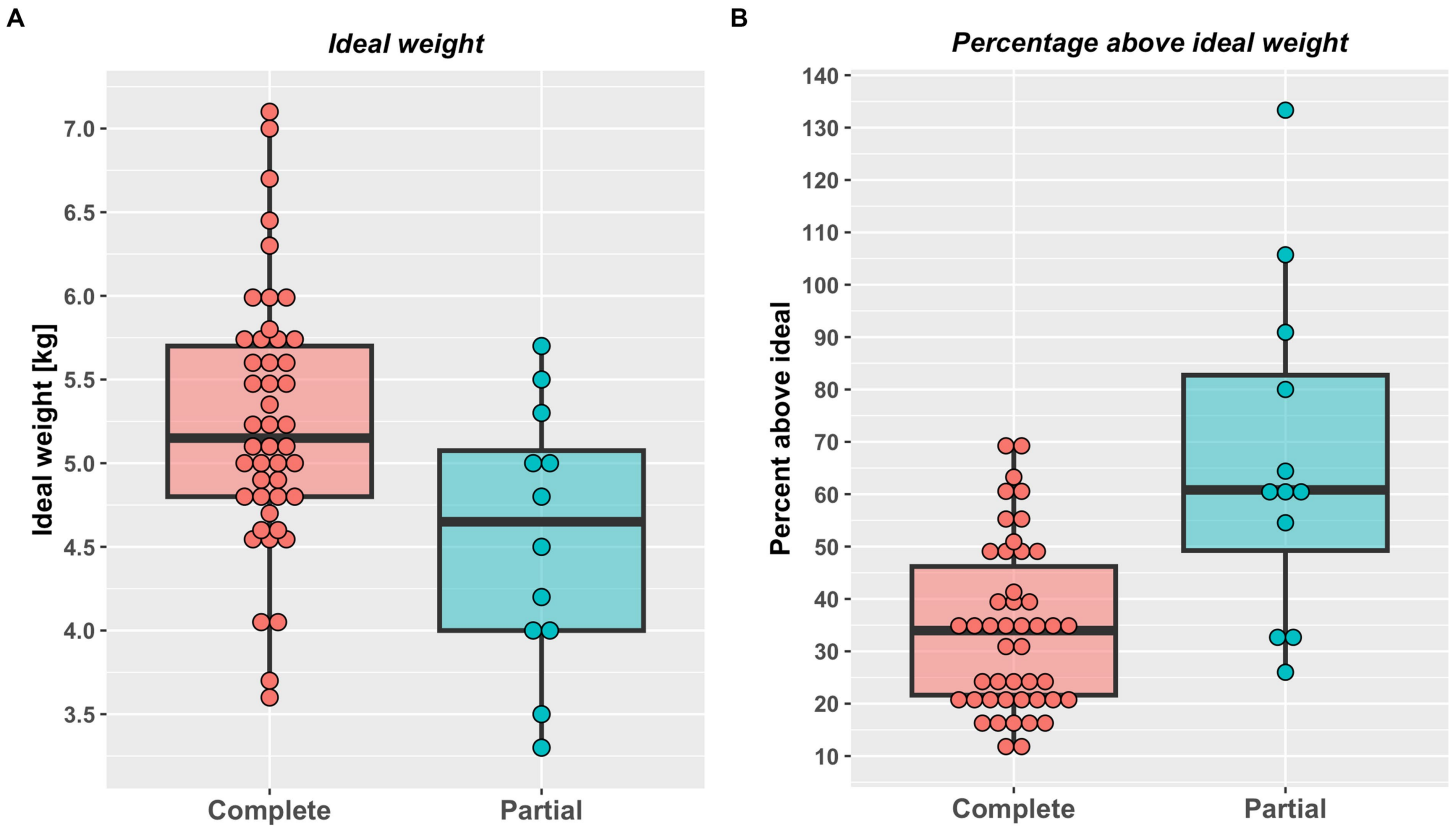
Estimated ideal weight in kg **(A)** and percentage above ideal weight **(B)** in cats undergoing either complete (46) or partial (12) weight reduction. Red (complete weight reduction protocol) or blue (partial weight reduction protocol) points represent data from individual cats, the thick horizontal black lines the median of each group, whilst the upper and lower hinges of the boxes represent the inter-quartile range (IQR). The lower whiskers represent the smallest observation greater than or equal to the lower hinge of the box minus 1.5 times the IQR; the upper whiskers represent the largest observation less than the upper hinge of the box plus 1.5 times the IQR. Both estimated ideal weight (complete 5.2 kg, 3.6–7.1 kg; partial 4.7 kg, 3.3–5.7 kg; *p* < 0.001, rank biserial −0.65 [very large effect], 95%-CI -0.82 to −0.39) and percentage above ideal weight less (complete: 34%, 11–69%; partial: 68%, 26–133%; *p* < 0.001, rank biserial −0.65 [very large effect], 95%-CI -0.82 to −0.39) were greater in cats undergoing complete weight reduction.

A total of 58 cats were included the study (Table 2). A complete weight reduction protocol was used in 46 cats (80%), with a partial weight loss protocol being used for the remaining 12 cats (20%), with the target weight being greater than ideal weight by a median of 18% (11–50%). There were no differences in sex, breed or starting bodyweight in cats that underwent complete versus partial weight reduction (Table 2). Not surprisingly, given the reasons for choosing the different protocols, cats assigned to complete weight reduction protocols were younger and percentage body fat was less than those assigned to partial weight reduction (Figure 1). There was no difference in starting weight between groups (Table 2), but estimated ideal weight was greater and percentage above ideal weight less in cats undergoing complete versus partial weight reduction (Figure 2; Table 2).

**Table 2:** Comparison of baseline data and outcomes in cats undergoing complete versus partial weight reduction.

*Variable*	Complete	Partial	*Between-protocol comparisons*	
			*p-*value^1^	*Rank biserial* ^2^
Number	46	12	–	–
Age (months)	86 (19–151)	130 (84–178)	**<0.001**	−0.70 (−0.84 to −0.46)^f^
Sex	29	9	0.515	–
*Neutered male*	17	3		
*Neutered female*				
Breed			0.142	–
*Domestic shorthair*	43	10		
*Domestic longhair*	2	0		
*Bengal cross*	0	1		
*Oriental longhair*	1	0		
*Maine Coon cross*	0	1		
Start weight (kg)	6.8 (4.4–10.3)	7.2 (6.3–10.5)	0.309	−0.19 (−0.51 to 0.17)^c^
Final weight (kg)	5.3 (3.7–7.4)	5.9 (3.7–7.2)	0.127	−0.29 (−0.58 to 0.07)^d^
Estimated ideal weight ^3^	5.2 (3.6–7.1)	4.7 (3.3–5.7)	**0.015**	0.46 (0.11 to 0.70)^f^
Percentage above ideal weight ^3^	34 (11–69)	68 (26–133)	**<0.001**	−0.65 (−0.82 to −0.39)^f^
Therapeutic diet used ^4^			0.251	–
*D1*	4	1		
*D2*	18	6		
*D1 and W1*	7	1		
*D2 and W1*	16	2		
*D2 and W2*	1	2		
Percentage dry food	78 (21–100)	76 (40–100)	0.560	0.11 (−0.26 to 0.45)^c^
Mean EI during weight reduction			0.589	−0.11 (−0.44 to 26)^c^
*KJ per kg^0.67^ per day*	222 (180–276)	222 (192–335)	–	–
*Kcal per kg^0.67^ per day*	53 (43–66)	53 (46–80)	–	–
Number of visits	11 (4–40)	7 (4–19)	**0.009**	0.50 (0.17 to 0.72)^f^
Duration (days)	294 (113–967)	178 (54–512)	0.153	0.27 (−0.09 to 0.57)^d^
Weight loss (%)^5^	23% (10–39%)	25% (10–41%)	0.886	−0.03 (−0.38 to 0.33)^a^
Rate of weight loss (% per week)^6^	0.61% (0.13–1.23%)	0.81% (0.41–1.36%)	**0.028**	−0.41 (−0.67 to −0.07)^f^

Data are expressed as median (range). ^1^*p*-values are from Mann–Whitney tests comparing complete with partial weight reduction. ^2^Effect size reported as rank biserial (with 95% confidence interval) and interpreted according to Funder and Ozer (19): <0.05, tiny^a^; 0.05–0.10, very small^b^; 0.10–0.20, small^c^; 0.20–0.30, medium^d^; 0.30–0.40, large^e^; >0.40 very large^f^. ^3^Estimated ideal weight and percentage above ideal weight was determined either by dual-energy X-ray absorptiometry or from body condition score (see methods). ^4^The therapeutic diets used were as follows: D1: dry diet 1; D2: dry diet 2; W1: wet diet 1; W2: wet diet 2; please see Table 1 legend for details of the specific therapeutic diets used. ^5^Percentage weight loss and rate of weight loss based on starting body weight. *p*-values <0.05 are indicated in bold.

### 3.2. Weight reduction outcomes

**Figure 3:**
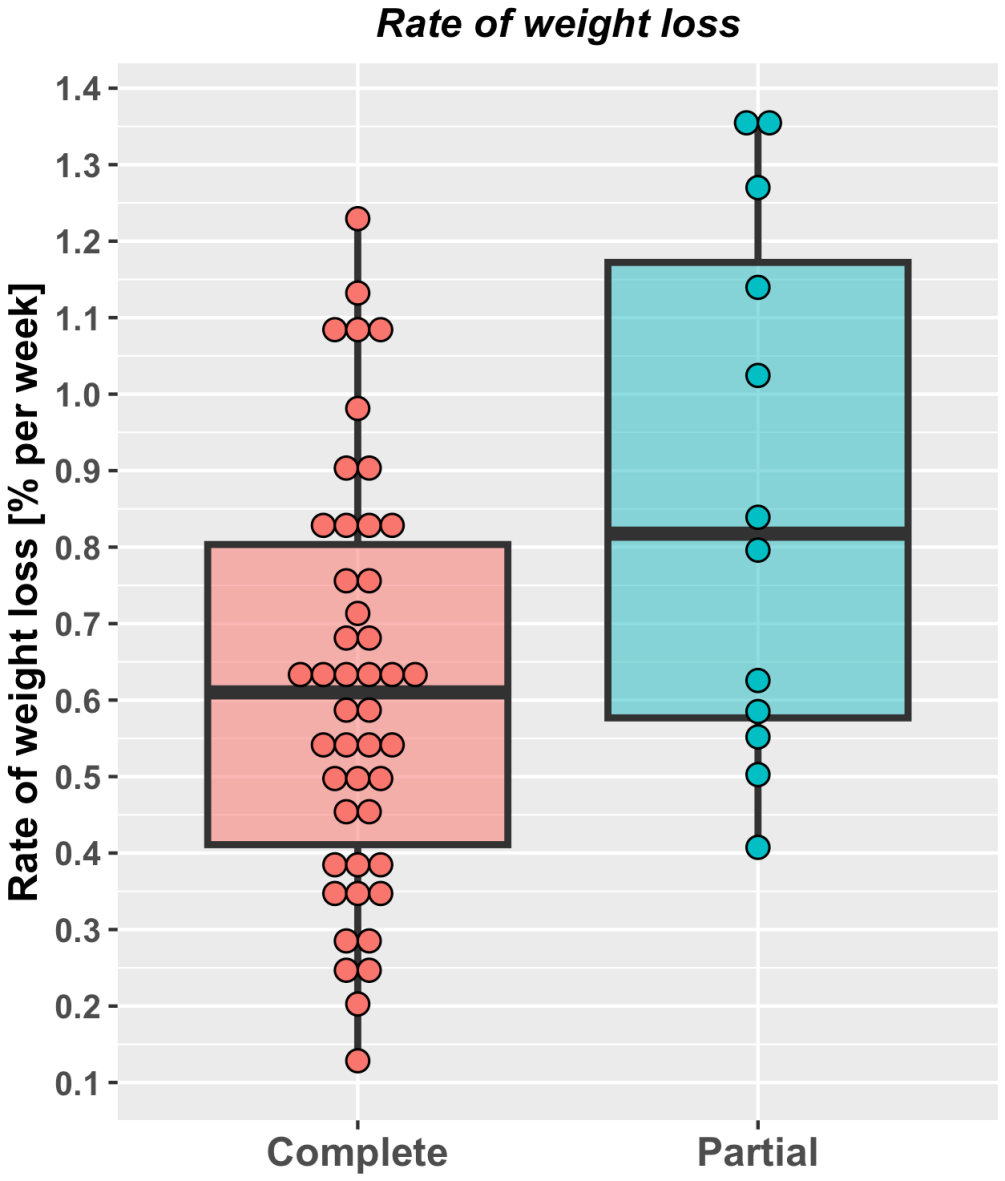
Rate of weight loss (% starting bodyweight per week) in cats undergoing either complete (46) or partial (12) weight reduction. Rate of weight loss for individual cats are represented by points, the solid horizontal black line represents the mean and upper and lower hinges of the boxes represent the inter-quartile range (IQR). The lower whisker represents the smallest observation greater than or equal to the lower hinge of the box minus 1.5 times the IQR; the upper whisker represents the largest observation less than the upper hinge of the box plus 1.5 times the IQR. Rate of weight loss was slower in cats that underwent complete [(0.61% SBW per week, 0.12–1.23%) compared with partial (0.82% SBW per week, 0.41–1.36%) weight reduction (*p* = 0.028, rank biserial −0.41, 95%-CI -0.67% to −0.07% (large effect size)].

Approximately half (29, 50%) the study cats were fed a dry therapeutic diet exclusively, whilst the rest were fed a combination of wet and dry therapeutic diets, with no differences in the therapeutic diets used, the proportion of dry food fed or number of visits between cats undergoing complete versus partial weight reduction protocols (Table 2). Overall percentage of weight lost was 23% (10–39%) and 25% (10–41%) over a median of 294 days (113 to 967 days) and 178 days (54 to 512 days), in cats that underwent complete or partial weight reduction, respectively, with no between-group differences. There was also no difference in median energy intake during the weight loss period between groups (Table 2). However, rate of weight loss was slower (Figure 3) and more visits were required (Table 2) in cats that underwent complete compared with partial weight reduction.

### 3.3. Changes in body composition during weight reduction

Details of body composition analysis in the 49 cats (complete weight reduction: 44 pre, 40 post; complete weight reduction: 9 pre, 7 post) were this was performed are shown in Table 3. Before weight reduction, there were no differences in both total mass (complete 6.35 kg, 3.98–9.91 kg; partial 6.78 kg, 5.80–10.03, *p* = 0.387, rank biserial −0.19, 95%-CI -0.54 to 0.22 [small effect]) and bone mineral content (complete 155 g, 96–221 g; partial 144 g, 104–201 g, *p* = 0.456, rank biserial 0.16, 95%-CI -0.25 to 0.52 [small effect]) between protocols; however, lean mass was greater (complete 4.20 kg, 2.64–5.72 kg; partial 3.45 kg, 2.79–4.71, *p* < 0.020, rank biserial 0.50, 95%-CI 0.13 to 0.75 [very large effect]), whilst both fat mass (complete 1.95 kg, 1.08–4.17 kg; partial 3.33 kg, 2.08–5.79 kg, *p* = 0.004, rank biserial −0.60, 95%-CI -0.80 to −0.27 [very large effect]) and fat percentage (complete 32%, 22–42%; partial 45%, 34–54%, *p* < 0.001, rank biserial −0.80, 95%-CI -0.91 to −0.59, [very large effect]) were less in cats that underwent complete versus partial weight reduction protocols.

**Table 3:** Body composition analysis in cats undergoing complete versus partial weight reduction.

*Component*	Complete	Partial	*Between-protocol comparisons*	
			*p-*value^1^	Rank biserial^2^
Total mass before (kg)^3^	6.35 (3.98–9.91)	6.78 (5.80–10.03)	0.387	−0.19 (−0.54 to 0.22)^c^
Total mass after (kg)^3^	4.80 (3.29–6.97)	5.50 (4.61–6.26)	0.150	−0.35 (−0.68 to 0.10)^e^
Within protocol comparisons				
*p-*value ^4^	**<0.001**	**0.016**		
Rank biserial	1.00 (1.00 to 1.00)^f^	1.00 (1.00 to 1.00)^f^		
Lean mass before (kg)	4.20 (2.64–5.72)	3.45 (2.79–4.71)	**0.020**	0.50 (0.13 to 0.75)^f^
Lean mass after (kg)	3.90 (2.76–5.24)	3.41 (2.90–4.59)	0.091	0.41 (−0.04 to 0.72)^f^
Within protocol comparisons				
*p-*value ^4^	**<0.001**	0.109		
Rank biserial	0.94 (0.88 to 0.97)^f^	0.71 (0.07 to 0.94)^f^		
Fat mass before (kg)	1.95 (108–4.17)	3.33 (2.08–5.79)	**0.004**	−0.60 (−0.80 to −0.27)^f^
Fat mass after (kg)	0.81 (0.20–1.60)	1.60 (1.27–2.46)	**<0.001**	−0.95 (−0.98 to −0.88)^f^
Within protocol comparisons				
*p-*value ^4^	**<0.001**	**0.016**		
Rank biserial ^2^	1.00 (1.00 to 1.00)^f^	1.00 (1.00 to 1.00)^f^		
Bone mineral content before (g)	155 (96–221)	144 (104–201)	0.456	0.16 (−0.25 to 0.52)^c^
Bone mineral content after (g)	141 (92–193)	123 (114–181)	0.491	0.17 (−0.29 to 0.57)^c^
Within protocol comparisons				
*p-*value ^4^	**<0.001**	**0.047**		
Rank biserial ^2^	1.00 (1.00 to 1.00)^f^	0.86 (0.43 to 0.97)^f^		
Fat mass before (%)	32% (22–42%)	45% (34–54%)	**<0.001**	−0.80 (−0.91 to −0.59)^f^
Fat mass after (%)	17% (5–25%)	31% (24–44%)	**<0.001**	−0.99 (−0.99 to −0.96)^f^
Within protocol comparisons				
*p-*value ^4^	**<0.001**	**0.016**		
Rank biserial ^2^	1.00 (1.00 to 1.00)^f^	1.00 (1.00 to 1.00)^f^		

Data are expressed as median (range). ^1^*p*-values are from Mann–Whitney tests comparing complete with partial weight reduction. ^2^Effect size reported as rank biserial (with 95% confidence interval) and interpreted according to Funder and Ozer (19): <0.05, tiny^a^; 0.05–0.10, very small^b^; 0.10–0.20, small^c^; 0.20–0.30, medium^d^; 0.30–0.40, large^e^; >0.40 very large^f^. ^3^Total mass represents the sum of lean mass, fat mass and bone mineral content as determined by dual-energy X-ray absorptiometry; please note that this measurement is always marginally less than mass determined by electronic weigh scales (see start weight and final weight in Supplementary Table S1). ^4^*p*-values are from Wilcoxon signed ranks tests comparing measurements taken before and after weight reduction within each protocol. *p*-values <0.05 are indicated in bold.

After weight reduction, again there were no differences in both total mass [complete 4.80 kg, 3.29–6.97 kg; partial 5.50 kg, 4.61–6.26, *p* = 0.150, rank biserial −0.35, 95%-CI -0.68 to 0.10, (medium effect) and bone mineral content (complete 141 g, 92–193 g; partial 123 g, 114–181 g, *p* = 0.491, rank biserial 0.17, 95%-CI -0.29 to 0.57 small effect) between protocols; however, both fat mass (complete 0.81 kg, 0.20–1.60 kg; partial 1.60 kg, 1.27–2.46 kg, *p* < 0.001, rank biserial −0.95, 95%-CI -0.98 to −0.88 (very large effect), and fat percentage (complete 17%, 22–40%; partial 31%, 24–44%, *p* < 0.001, rank biserial −0.99, 95%-CI -0.99 to −0.96 (very large effect) were less in cats that underwent complete versus partial weight reduction protocols, whilst there was no difference in lean mass between protocols (complete 3.90 kg, 2.76–5.24 kg; partial 3.41 kg, 2.90–4.59 kg, *p* = 0.092, rank biserial 0.41, 95%-CI -0.04 to 0.72 (very large effect)].

When within-protocol changes in body composition during weight reduction were assessed, there were significant decreases were observed in total mass, fat mass, bone mineral content, and fat percentage for cats on either complete or partial protocols (Table 3). However, although lean mass decreased significantly in cats undergoing complete weight reduction (before 4.20 kg, 2.64–5.72 kg; after 3.90 kg, 2.76–5.24 kg, *p* < 0.001, rank biserial 0.94, 95%-CI 0.88–0.97 [very large effect]), there was no change in lean mass for cats undergoing partial weight reduction (before 3.45 kg, 2.79–4.71 kg; after 3.41 kg, 2.90–4.59 kg, *p* = 0.109, rank biserial 0.71, 95%-CI 0.07–0.94 [very large effect]).

### 3.4. Estimation of essential nutrient intake during weight reduction

Average daily essential nutrient intakes during weight reduction were compared with both NRC 2006 and FEDIAF recommendations (Table 4). Average intake of all nutrients was greater than both NRC and FEDIAF recommendations, besides choline, phenylalanine/tyrosine, potassium and selenium. With selenium, average daily intake was under NRC AI and RA in 42 cats (72%; 33/46 complete [72%], 9/12 partial [75%]), and less than FEDIAF recommendation in 33 cats (57%; 25/46 complete [54%], 8/12 partial [8%]). Average daily intake of choline was under NRC MR, NRC RA and FEDIAF recommendation in 22 (38%; 16/46 complete [35%], 6/12 partial [50%]), 53 (91%, 44 complete [92%], 9 partial [75%]) and 51 (88%; 42 complete [91%], 9 partial [75%]) cats, respectively. In 2 (3%; 2 complete [5%], 0 partial [0%]), 2 (3%, 2 complete [5%], 0 partial [0%]) and 8 (14%; 7/46 complete [15%], 1/12 partial [8%]) cats, average daily intake of potassium was marginally (i.e., within 10% of the recommendation) less than NRC AI, NRC RA and FEDIAF recommendation, respectively. Finally, the intake of phenylalanine and tyrosine combined was marginally (i.e., within 5% of the recommendation) less than NRC AI, NRC RA and FEDIAF recommendation in 7 (12%; complete: 5/46 [10%], partial: 2/12 [17%]), 7 (12%, complete: 5/46 [10%], partial: 2/12 [17%]) and 8 (14%; complete: 6/46 [13%], partial: 2/12 [17%]) cats, respectively. Essential nutrient intakes did not differ between cats undergoing complete or partial weight reduction (*p* > 0.500 for all; effect sizes either tiny or very small; Table 5).

**Table 4:** Average daily essential nutrient intake during weight reduction in 53 cats with obesity.

***Nutrient***	NRC recommendation^1^			FEDIAF^b^	Median (range)	Number (%) below NRC^1^			FEDIAF^2^
	MR	AI	RA			MR	AI	RA	
*Arginine (g)*	–	0.19	0.19	0.25	0.33 (0.27–0.45)	–	0 (0)	0 (0)	0 (0)
*Histidine (g)*	–	0.064	0.064	0.065	0.122 (0.098–0.169)	–	0 (0)	0 (0)	0 (0)
*Isoleucine (g)*	–	0.11	0.11	0.11	0.22 (0.18–0.30)	–	0 (0)	0 (0)	0 (0)
*Methionine (g)*	0.033	–	0.042	0.43	0.116 (0.091–0.160)	0 (0)	–	0 (0)	0 (0)
*Met and cys (g) ^3^*	0.067	–	0.084	0.085	0.204 (0.159–0.279)	0 (0)	–	0 (0)	0 (0)
*Leucine (g)*	–	0.25	0.25	0.26	0.45 (0.38–0.63)	–	0 (0)	0 (0)	0 (0)
*Lysine (g)*	0.067	–	0.084	0.085	0.268 (0.188–0.385)	0 (0)	–	0 (0)	0 (0)
*Phenylalanine (g)*	–	0.099	0.099	0.1000	0.250 (0.203–0.346)	–	0 (0)	0 (0)	0 (0)
*Phe and Tyr (g) ^4^*	–	0.38	0.38	0.38	**0.44 (0.36–0.60)**	**–**	**7 (12)**	**7 (12)**	**8 (14)**
*Threonine (g)*	–	0.13	0.13	0.13	0.22 (0.18–0.30)	–	0 (0)	0 (0)	0 (0)
*Tryptophan (g)*	–	0.032	0.032	0.033	0.058 (0.046–0.082)	–	0 (0)	0 (0)	0 (0)
*Valine (g)*	–	0.13	0.13	0.128	0.27 (0.22–0.38)	–	0 (0)	0 (0)	0 (0)
*Taurine (g) ^5^*	0.0079	–	0.0099	0.050 / 0.025	0.0529 (0.0288–0.1186)	0 (0)	–	0 (0)	0 (0)
*Linoleic acid (g)*	–	0.14	0.14	0.13	0.30 (0.24–0.44)	–	0 (0)	0 (0)	0 (0)
*Arachidonic acid (g)*	–	0.0005	0.0015	0.0015	0.0157 (0.0112–0.0286)	–	0 (0)	0 (0)	0 (0)
*EPA and DHA (g) ^6^*	–	0.0025	0.0025	–	0.0239 (0.0109–0.0350)	–	0 (0)	0 (0)	0 (0)
*Calcium (g)*	0.040	–	0.071	0.100	0.222 (0.160–0.303)	0 (0)	–	0 (0)	0 (0)
*Phosphorus (g)*	0.035	–	0.063	0.064	0.190 (0.145–0.254)	0 (0)	–	0 (0)	0 (0)
*Magnesium (mg)*	4.9	–	9.5	10.0	13.2 (10.6–17.5)	0 (0)	–	0 (0)	0 (0)
*Sodium (mg)*	16.0	–	16.7	19.0	98.9 (64.7–147.3)	0 (0)	–	0 (0)	0 (0)
*Potassium (g)*	–	0.13	0.13	0.15	**0.17 (0.12–0.23)**	**–**	**2 (3)**	**2 (3)**	**8 (14)**
*Chloride (mg)*	–	23.7	23.7	29.0	178.6 (119.7–231.7)	–	0 (0)	0 (0)	0 (0)
*Iron (mg)*	–	1.98	1.98	2.00	2.89 (2.12–3.83)	–	0 (0)	0 (0)	0 (0)
*Copper (mg)*	–	0.119	0.119	0.125	0.286 (0.208–0.407)	–	0 (0)	0 (0)	0 (0)
*Zinc (mg)*	–	1.9	1.9	1.9	3.0 (2.2–3.9)	–	0 (0)	0 (0)	0 (0)
*Manganese (mg)*	–	0.119	0.119	0.125	0.937 (0.459–1.354)	–	0 (0)	0 (0)	0 (0)
*Selenium (μg)*	–	6.95	6.95	6.50	**6.35 (4.61–8.92)**	**–**	**42 (72)**	**42 (72)**	**33 (57)**
*Iodine (μg)*	31.6	–	35.0	33.0	56.8 (41.7–77.6)	0 (0)	–	0 (0)	0 (0)
*Vitamin A (RE) ^7^*	–	19.8	24.7	25.0	319.8 (122.6–1024.9)	–	0 (0)	0 (0)	0 (0)
*Cholecalciferol (μg)*	–	0.14	0.17	0.16	0.43 (0.31–0.63)	–	0 (0)	0 (0)	0 (0)
*Vitamin E (mg)*	–	0.74	0.94	0.64	10.83 (7.46–15.13)	–	0 (0)	0 (0)	0 (0)
*Thiamine (mg)*	–	0.11	0.14	0.11	0.54 (0.25–1.56)	–	0 (0)	0 (0)	0 (0)
*Riboflavin (mg)*	–	0.079	0.099	0.080	0.891 (0.369–1.346)	–	0 (0)	0 (0)	0 (0)
*Pyridoxine (mg)*	0.05	–	0.06	0.14	0.67 (0.30–0.99)	0 (0)	–	0 (0)	0 (0)
*Niacin (mg)*	–	0.79	0.99	0.80	3.14 (2.15–4.39)	–	0 (0)	0 (0)	0 (0)
*Pantothenic acid (mg)*	0.11	–	0.14	0.14	0.97 (0.60–1.37)	0 (0)	–	0 (0)	0 (0)
*Cobalamin (μg)*	–	0.44	0.56	0.44	2.59 (1.41–3.71)	–	0 (0)	0 (0)	0 (0)
*Folic acid (μg)*	15	–	19	29	306 (125–464)	0 (0)	–	0 (0)	0 (0)
*Biotin (mg)*	–	1.5	1.9	1.5	48.7 (19.6–74.2)	–	0 (0)	0 (0)	0 (0)
*Choline (mg)*	50	–	63	60	**52 (39–69)**	**22 (38)**	**–**	**53 (91)**	**51 (88)**

^1^Figures for each nutrient reported as stated units per kg^0.67^ of ideal bodyweight per day. Please note that figures are reported to the same number of decimal places as either the adequate intake (AI), minimal requirement (MR) and recommended allowance (RA) as listed in the National Research Council report (8). ^2^Nutritional requirements recommended by the Fédération Européenne de l’Industrie des Aliments pour Animaux Familiers (8); please note that the figures reported have been converted from SI units to US units to facilitate comparison with the NRC 2006 requirements. ^3^Met and cys: methionine and cysteine. ^4^Phe and tyr: phenylalanine and tyrosine. ^5^Please note that FEDIAF make separate recommendations for dietary taurine depending on whether wet or dry food; for cats in the current study, estimated taurine intake was compared with an individualized FEDIAF recommendation for that cat based on the proportion of wet and dry food consumed. ^6^EPA and DHA: eicosapentanoic acid and docosahexaenoic acid; ^7^RE: retinol equivalents. Results are highlighted in bold for nutrients where intakes, in some cats, were less than one or more of the thresholds assessed (NRC 2006 AI, RA or MR, FEDIAF).

**Table 5:** Comparison of essential nutrient intakes during weight reduction in cats undergoing complete versus partial weight reduction.

*Nutrient*	Complete weight reduction	Partial weight reduction	*p-*value^1^	Rank biserial^2^
*Arginine (g)*	0.33 (0.27–0.44)	0.32 (0.27–0.45)	0.755	−0.06 (−0.41 to 0.30)^a^
*Histidine (g)*	0.124 (0.98–0.161)	0.119 (0.098–0.169)	0.615	−0.10 (−0.44 to 0.27)^b^
*Isoleucine (g)*	0.22 (0.18–0.30)	0.21 (0.18–0.30)	0.917	−0.02 (−0.37 to 0.33)^a^
*Methionine (g)*	0.116 (0.091–0.149)	0.117 (0.091–0.160)	0.615	−0.10 (−0.44 to 0.27)^b^
*Met and cys (g)^3^*	0.205 (0.159–0.270)	0.203 (0.159–0.279)	0.615	−0.10 (−0.44 to 0.27)^b^
*Leucine (g)*	0.46 (0.38–0.63)	0.44 (0.38–0.62)	0.977	0.00 (−0.35 to 0.36)^a^
*Lysine (g)*	0.266 (0.188–0.380)	0.276 (0.188–0.385)	0.684	−0.08 (−0.42 to 0.28)^b^
*Phenylalanine (g)*	0.252 (0.203–0.325)	0.245 (0.212–0.346)	0.813	−0.05 (−0.39 to 0.31)^b^
*Phe and Tyr (g)^4^*	0.45 (0.36–0.58)	0.43 (0.37–0.60)	0.789	−0.05 (−0.40 to 0.31)^b^
*Threonine (g)*	0.22 (0.18–0.28)	0.21 (0.18–0.30)	0.589	−0.11 (−0.44 to 0.26)^c^
*Tryptophan (g)*	0.058 (0.046–0.077)	0.058 (0.046–0.082)	0.642	−0.09 (−0.43 to 0.27)^b^
*Valine (g)*	0.28 (0.22–0.36)	0.26 (0.22–0.38)	0.623	−0.09 (−0.43 to 0.27)^b^
*Taurine (g)^5^*	0.0529 (0.0288–0.1186)	0.0568 (0.0367–0.0956)	0.813	−0.05 (−0.39 to 0.31)^b^
*Linoleic acid (g)*	0.30 (0.25–0.41)	0.30 (0.024–0.44)	0.917	−0.02 (−0.37 to 0.33)^a^
*Arachidonic acid (g)*	0.0155 (0.0112–0.0259)	0.0168 (0.0112–0.0286)	0.589	−0.11 (−0.44 to 0.26)^c^
*EPA and DHA (g) ^6^*	0.0234 (0.0109–0.0322)	0.0247 (0.0147–0.0350)	0.769	−0.06 (−0.40 to 0.30)^b^
*Calcium (g)*	0.222 (0.160–0.259)	0.217 (0.183–0.303)	0.977	0.00 (−0.35 to 0.36)^a^
*Phosphorus (g)*	0.190 (0.145–0.235)	0.191 (0.156–0.254)	0.932	−0.02 (−0.37 to 0.34)^a^
*Magnesium (mg)*	13.2 (10.6–17.5)	13.2 (10.6–17.4)	0.902	−0.03 (−0.38 to 0.33)^a^
*Sodium (mg)*	98.9 (64.7–132.2)	100.1 (83.7–147.3)	0.602	−0.10 (−0.44 to 0.26)^b^
*Potassium (g)*	0.17 (0.12–0.21)	0.17 (0.14–0.23)	0.992	0.00 (−0.36 to 0.35)^a^
*Chloride (mg)*	179.8 (119.7–212.8)	173.5 (147.1–231.7)	0.842	−0.04 (−0.39 to 0.32)^a^
*Iron (mg)*	2.90 (2.12–3.42)	2.83 (2.36–3.83)	0.917	−0.02 (−0.37 to 0.33)^a^
*Copper (mg)*	0.286 (0.208–0.359)	0.286 (0.247–0.407)	0.712	−0.07 (−0.42 to 0.29)^b^
*Zinc (mg)*	3.0 (2.2–3.6)	2.9 (2.4–3.9)	0.887	−0.03 (−0.38 to 0.33)^a^
*Manganese (mg)*	0.918 (0.459–1.261)	0.975 (0.589–1.354)	0.755	−0.06 (−0.41 to 0.30)^b^
*Selenium (mg)*	6.33 (4.61–7.85)	6.37 (5.52–8.92)	0.726	−0.07 (−0.41 to 0.29)^b^
*Iodine (mg)*	56.8 (41.7–72.3)	56.5 (42.0–77.6)	0.726	−0.07 (−0.41 to 0.20)^b^
*Vitamin A (RE) ^7^*	319.8 (122.6–1024.9)	337.3 (126.6–758.9)	0.513	−0.13 (−0.46 to 0.24)^c^
*Cholecalciferol (μg)*	0.42 (0.31–0.52)	0.43 (0.37–0.63)	0.784	−0.05 (−0.40 to 0.31)^b^
*Vitamin E (mg)*	10.83 (7.46–14.09)	10.99 (7.83–15.13)	0.698	−0.08 (−0.42 to 0.29)^b^
*Thiamine (mg)*	0.54 (0.25–1.56)	0.62 (0.25–1.18)	0.513	−0.13 (−0.46 to 0.24)^c^
*Riboflavin (mg)*	0.877 (0.369–1.253)	0.966 (0.538–1.346)	0.887	−0.03 (−0.38 to 0.33)^a^
*Pyridoxine (mg)*	0.66 (0.30–0.92)	0.71 (0.41–0.99)	0.842	−0.04 (−0.39 to 0.32)^a^
*Niacin (mg)*	3.14 (2.15–4.09)	3.19 (2.26–4.39)	0.698	−0.08 (−0.42 to 0.29)^b^
*Pantothenic acid (mg)*	0.97 (0.60–1.28)	0.99 (0.67–1.37)	0.684	−0.08 (−0.42 to 0.28)^b^
*Cobalamin (μg)*	2.56 (1.41–3.45)	2.68 (1.69–3.71)	0.755	−0.06 (−0.41 to 0.30)^b^
*Folic acid (μg)*	302 (125–432)	333 (185–464)	0.902	−0.03 (−0.38 to 0.33)^a^
*Biotin (mg)*	48.0 (19.6–69.1)	53.2 (29.3–74.2)	0.917	−0.02 (−0.37 to 0.33)^a^
*Choline (mg)*	52 (39–64)	51 (41–69)	0.872	−0.03 (−0.38 to 0.33)^a^

Figures for each nutrient reported as median (range) stated units per kg^0.67^ of ideal bodyweight per day. Please note that figures are reported to the same number of decimal places as listed in the National Research Council report (7). See the legend to Table. ^1^*p*-values are from Mann–Whitney tests comparing complete with partial weight reduction. ^2^Effect size reported as rank biserial (with 95% confidence interval) and interpreted according to Funder and Ozer (19): <0.05, tiny^a^; 0.05–0.10, very small^b^; 0.10–0.20, small^c^; 0.20–0.30, medium^d^; 0.30–0.40, large^e^; >0.40 very large^f^. ^3^Met and cys: methionine and cysteine. ^4^Phe and tyr: phenylalanine and tyrosine. ^5^Please note that FEDIAF make separate recommendations for dietary taurine depending on whether wet or dry food; for cats in the current study, estimated taurine intake was compared with an individualised FEDIAF recommendation for that cat based on the proportion of wet and dry food consumed. ^6^EPA and DHA: eicosapentanoic acid and docosahexaenoic acid; ^7^RE: retinol equivalents.

## 4. Discussion

In this study, we have compared differences in outcomes and in essential nutrient intake in cats undergoing either partial or complete weight reduction using purpose-formulated therapeutic diets. Even though the cats undergoing partial weight reduction were older and had a greater body fat percentage, some weight loss outcomes (such as rate of weight loss per week) were better, whilst there was no difference in outcome for many others. These results are encouraging since marked obesity is known to be negatively associated with outcomes of weight reduction in cats (11). Therefore, offering a partial weight reduction protocol might be a suitable compromise in such cases to maximize the chances of a successful outcome.

Prior to the weight reduction period, some notable differences were evident between the cats in the two groups: compared with cats where partial weight reduction was chosen, those allocated to a complete weight reduction protocol were younger, on average, but their fat mass and percentage fat were less. These differences are to be expected because of the criteria where age and degree of obesity were key variables on which decision-making was based. Despite the lesser fat mass, estimated ideal weight was greater in cats assigned to complete weight reduction protocols; body composition data revealed this to be the result of lean tissue mass being greater in those assigned to complete, compared with partial, weight reduction protocols. The reason for this difference is not known, but older age could be a key factor. In human beings, lean tissue mass declines during most of adult life, with the average male having ~12 kg less lean mass at age 65–70 compared with age 25 (20). Such declines in lean mass are related to poor health and disability in older people (21), whilst lean mass is negatively associated with mortality risk (22–24). There have been relatively few studies examining changes in lean mass during ageing in dogs (reviewed by Freeman, 2011) (25) where, generally, an age-related decline in lean mass is observed (26, 27). Studies in cats are even more limited, with one study suggesting little association between lean mass and age in this species (28); however, in that study, none of the cats were older than 10 years’ age. Therefore, further work is required to determine the actual effect of increasing age on muscle mass in cats.

Although fat mass changes were similar between the groups, there were differences in lean tissue change, with lean mass being better preserved in those undergoing partial reduction. This finding should be interpreted cautiously because body composition was not performed in all cats, and relatively few were assigned to a partial weight reduction protocol. It might be that the non-significant difference in lean mass before and after weight reduction was because this analysis was underpowered, from a statistical point of view, and this is supported by the fact that the effect size was still classed as ‘very large’, despite the lack of statistical significance. An alternative explanation would be that, whilst loss of some lean tissue was observed in cats undergoing partial weight reduction protocols (lean mass before: 3.45 kg; lean mass after 3.41 kg), the magnitude of loss was greater in cats undergoing complete weight reduction (lean mass before: 4.20 kg; lean mass after: 3.90 kg). Overall, therefore, whilst these results tentatively suggest that lean tissue mass might better be preserved during a partial weight reduction protocol, further studies would be required to confirm this finding.

Besides possible benefits with preserving lean tissue mass, cats undergoing partial weight reduction protocols lost weight faster on average. Given that faster rates of weight loss are positively associated with a favorable outcome (i.e., reaching target weight) in cats with obesity (2, 11), this faster rate could be seen as an advantage of recommending partial rather than complete weight reduction. Added to this, fewer visits were required, which again could be advantageous given that the burden on owners would be less. However, to the authors’ knowledge, no studies have looked at associations between visit frequency and success of weight management in either cats or dogs.

A further aim of the study was to assess essential nutrient intakes during weight reduction, and to determine whether there were any differences in the intake of essential nutrients between cats undergoing complete and partial weight loss protocols. Overall, daily recommended intakes were comfortably met for most essential nutrients and, where requirements were not met, intake was close to recommendations. Further, all cats remained healthy throughout this weight reduction period and did not show any signs of nutrient deficiency. This suggests that, from a nutritional perspective, controlled weight reduction in cats with obesity is safe overall. These results extend those of other recent studies in cats (9, 10), but there were some differences, most notably that a larger cohort of cats was studied, some of which underwent a partial weight reduction protocol; further, in some of the cats undergoing a complete weight reduction protocol, there was marked weight loss (of up to 41% of starting body weight) over a prolonged duration (of up to 967 days). In contrast, Keller et al. (9) used reduced-energy maintenance diets for weight reduction for median duration of 50 days (49–63 days), with cats losing 4.5% SBW (−2.0 to 18.8% SBW) whilst the duration of weight reduction in the Grant et al. study (10) was 10 weeks (70 days), with cats losing an average of 9.4% SBW. Therefore, as well as the current study being larger, it was also more complete in terms of the variability in weight reduction outcomes that can be seen in pet cats with obesity that undergo controlled weight reduction.

Intake of choline was less than NRC RA and FEDIAF requirement in most cats, by a median of 17 and 13%, respectively, but was greater than NRC MR in over 60%. These findings mirror previous findings from a study where a different therapeutic diet was used, where all cats had an average choline intake under RA, and half the cats had a choline intake under MR (10). In cats, choline is required for cell membrane structure, neurotransmission, methyl metabolism, coagulation and hepatic lipid metabolism (7, 29). When fed at suboptimal concentrations, it is reported to depress growth in kittens (30–32) as well as leading to hypoalbuminemia and hepatic lipid accumulation (32). In the current study, and similar to previous work (10), all cats were healthy during the weight reduction protocol, and no signs of deficiency were seen. This might be because choline is not a true vitamin but a vitamin-like substance, not least because many animals can synthesize choline in the liver through methylation of ethanolamine (7). Therefore, although the NRC (7) suggest that diets should be formulated to include at least 637 mg of choline per 1,000 kcal, to meet the requirements of cats at all life stages, previous work has indicated that a choline-deficient diet can made adequate if methionine is supplemented beyond its requirements, because the liver can utilize methionine to synthesize choline *de novo* (33). The dietary methionine intake for the cats in the current study was estimated to be at least twice all recommendations; assuming these cats did not require methionine to synthesize cysteine (given that the combined intake of both was at least 80% greater than all recommendations), this amount was likely to be more than sufficient to compensate for any marginal intake of choline.

An additional consideration concerning choline requirements is whether requirements differ during a period of weight reduction, compared with maintenance. In a recent study, untargeted metabolomic techniques were used to analyze serum metabolite changes associated with weight reduction in overweight cats (29); a total of 269 metabolites were altered, with over half being associated with lipid metabolism, including choline that declined within the first week of weight reduction and remained lower throughout the study. Such a rapid decline is more likely to be consistent with altered metabolism rather than depletion of choline reserves arising from choline deficiency. Therefore, choline requirements might be less for cats during controlled weight reduction than when fed at maintenance. Nonetheless, the optimal intake of choline during weight reduction requires further investigation, not least because recent studies have shown possible beneficial effects of choline supplementation on hepatic fat mobilization for cats with obesity fed at maintenance requirements (34).

Regarding minerals, daily intake of selenium was less than NRC recommendations (both AI and RA) in most study cats. Given that intake of minerals was not reported in the Grant et al. study (10), the selenium status during weight loss for those cats is not known. However, selenium intake was assessed in the Keller et al. (9) study, with 4 of 17 cats having estimated intakes less than NRC AI recommendations. Pet food regulators have set limits on the amount of selenium that can be added to pet food (8), making it difficult to meet recommendations (16). This is particularly the case in Europe where the FEDIAF limit is particularly strict, on account of the added intention of decreasing environmental pollution with trace elements. That said, and as with choline intake, the significance of this finding is not known, not least given that none of the cats showed any signs of selenium deficiency; further, selenium works in synergy with vitamin E, so a moderate deficiency would be compensated for by this nutrient (7). Potassium was the only other mineral where, in a minority of cats, intake was slightly under recommendations. Whilst potassium intake was less than the FEDIAF recommendation in 8 cats (14%) by a maximum of 20%, it was less than both NRC recommendations in only 2 cats (3%), with the maximum shortfall being 5%. In a previous long-term feeding trial in cats, potassium deficiency was induced with the main clinical consequences being increased serum creatinine concentration and fractional excretion of potassium, suggesting possible renal compromise (35). Although fractional excretion of potassium was not measured in the current study, none of the cats developed azotemia or signs of chronic kidney disease. Therefore, the borderline potassium intakes in some cats are unlikely to be of significance.

Intake of most essential amino acids comfortably exceeded NRC recommendations in all cats of the current study, except for the combined intake of phenylalanine and tyrosine which was marginally less than recommendations in 12–14% of cats, depending on the recommendation used (NRC MA vs. NRC RA vs. FEDIAF). These findings contrast with previous work where, arginine intake did not meet NRC recommendations in all 16 cats studied (10). Although there might be various reasons for these differences, including differences in the study groups and study methodology, it is most likely to be due to the fact that energy restriction was slightly greater in the previous study and a different therapeutic diet was used, which contained less than half the amount of arginine (2.3 g per 1,000 Kcal) than was included in the therapeutic diets of the current study (6.4, 5.9 and 6.7 g per 1,000 Kcal for the HPD, HPHFD and HPW diets, respectively). The significance of the marginally low combined phenylalanine and tyrosine concentrations observed in the current study is not known, not least given that the intakes in question were only 5% less than the recommendations, whilst intake of phenylalanine alone was over twice that of all recommendations. In contrast to phenylalanine, which is an essential amino acid, tyrosine is not although its inclusion can spare the amount of phenylalanine that is required (7). Neither phenylalanine nor tyrosine are thought to be limiting amino acids for optimal nitrogen balance, although they are needed for the synthesis of thyroid hormones and catecholamines (7) and to produce maximal black hair color in kittens (36, 37); when intake of phenylalanine and tyrosine was suboptimal, a reddish-brown hair coat was observed (36). Signs of neurological dysfunction have also been observed when suboptimal intake occurred in kittens, manifesting as an uncoordinated gait, hyperactivity, hypersalivation and vocalization (7). No neurological or dermatological signs, including altered haircoat color were observed in any of the cats at any point during their weight reduction protocols.

There are several limitations to consider besides those already mentioned above. First, the study population was from a specialist referral obesity clinic and, therefore, it might not be possible to generalize results to all pet cats with obesity undergoing weight management. Second, the study groups were unbalanced with relatively few cats being enrolled on partial weight reduction protocols. Given that statistically significant differences were identified., both within and between groups, it is likely that, for many variables, the study had sufficient statistical power. However, we cannot exclude the possibility that the some genuine between-and within-group differences might have been missed if such differences were small. Therefore, ideally, the results of the current study should be confirmed in a future study where group sizes are larger.

A third limitation is the fact that only four therapeutic diets therapeutic diets were used from a single pet food manufacturer. The findings might have differed had other diets been used, as suggested by the differences between the current study and that of previous work (9, 10). A further limitation relates to the accuracy of food intake information and, therefore, the essential nutrient intakes. The cats studied were client-owned and lived at home throughout their period of weight loss; further, we relied on owner to measure food portions accurately and accurately record the amount their cat ate. It is possible that mistakes might have been introduced at this stage, not least given that previous research has demonstrated both mis-and under-reporting of food intake in human nutrition studies (38). Further, given that some cats had outdoor access, they might have been able to access food from other food sources (e.g., hunting or food from neighbors) without their owners knowing. A final limitation is that the actual essential nutrient requirements for cats with obesity during controlled weight reduction are not known; instead, we had to use guidelines that are for cats fed at MER. For some nutrients, essential requirements might be the same as maintenance requirements, other requirements might increase, whilst some might decrease because of physiological changes occurring during weight management. Further work would be required to confirm exact requirements in such a situation.

## 5. Conclusion

Partial weight reduction protocols can successfully induce weight loss in cats, including both cats with severe obesity and those that are older, possibly, minimizing the amount of lean tissue lost. Further studies are required to extend these observations. Major essential nutrient deficiencies did not occur when feeding purpose-formulated therapeutic diets either for partial or complete weight reduction protocols for cats with obesity. Although intakes of selenium and choline were often borderline-low, the fact that all cats remained healthy throughout, and knowledge that other nutrients can compensate for such intakes, suggest that weight reduction using therapeutic diets is safe overall.

## Data availability statement

The original contributions presented in the study are included in the article/Supplementary material, further inquiries can be directed to the corresponding author.

## Ethics statement

The animal study was reviewed and approved by University of Liverpool Veterinary Research Ethics Committee. Written informed consent was obtained from the owners for the participation of their animals in this study.

## Author contributions

AG: conceptualization, methodology, formal analysis, data curation, validation, writing – original draft preparation, writing – review and editing, project administration, funding acquisition, resources, and visualization. GW-L: data curation, validation, writing – review and editing, and project administration. VB: conceptualization, validation, writing – review and editing, and funding acquisition. JF: conceptualization, writing – review and editing, and funding acquisition. All authors contributed to the article and approved the submitted version.

## Funding

The study was funded by grants from Royal Canin (VCR10030, JXR10085, JAXR10461 and JXR31196), a division of Mars Petcare, and this company manufactured the diets fed in this study.

## Conflict of interest

VB and JF are employees of Royal Canin. AG and GW-L are employees of the University of Liverpool but their positions are funded by Royal Canin. Both have received financial remuneration and gifts for providing educational material, speaking at conferences, and consultancy work.

## Publisher’s note

All claims expressed in this article are solely those of the authors and do not necessarily represent those of their affiliated organizations, or those of the publisher, the editors and the reviewers. Any product that may be evaluated in this article, or claim that may be made by its manufacturer, is not guaranteed or endorsed by the publisher.
